# Supplementary material for: Filamin A organizes γ‑aminobutyric acid type B receptors at the plasma membrane
Source: Nat Commun. 2023 Jan 3;14:34. doi: 10.1038/s41467-022-35708-1 (PMC9810740; doi:10.1038/s41467-022-35708-1)

**Supplementary Information**

**Filamin A organizes γ‑aminobutyric acid type B receptors at the plasma membrane**

Marie-Lise Jobin^1,2#*^, Sana Siddig^1,2^, Zsombor Koszegi^3,4^, Yann Lanoiselée^3,4^, Vladimir Khayenko^5^, Titiwat Sungkaworn^1,2^, Christian Werner^6^, Kerstin Seier^1,2^, Christin Misigaiski^1,2^, Giovanna Mantovani^7,8^, Markus Sauer^6^, Hans M. Maric^5^, Davide Calebiro^1,2,3,4*^

^1^ Institute for Pharmacology and Toxicology, University of Würzburg, Würzburg, Germany

^2^ Bio-Imaging Center/Rudolf Virchow Center, University of Würzburg, Würzburg, Germany

^3^ Institute of Metabolism and Systems Research, University of Birmingham, Birmingham, UK

^4^ Centre of Membrane Proteins and Receptors (COMPARE), Universities of Birmingham and Nottingham, UK

^5^ Rudolf Virchow Center, Center for Integrative and Translational Bioimaging, University of Würzburg, Würzburg, Germany

^6^ Department of Biotechnology and Biophysics, Biocenter, University of Würzburg, Würzburg, Germany

^7^ Endocrinology Unit, Fondazione IRCCS Ca' Granda Ospedale Maggiore Policlinico, Milan, Italy

^8^ Department of Clinical Sciences and Community Health, University of Milan, Milan, Italy

^#^ Present address: Interdisciplinary Institute for Neuroscience (IINS), CNRS UMR5297, University of Bordeaux, 33000 Bordeaux, France

* Corresponding authors: Marie-Lise Jobin ([marie-lise.jobin@u-bordeaux.fr](mailto:marie-lise.jobin@u-bordeaux.fr)), Davide Calebiro ([D.Calebiro@bham.ac.uk](mailto:D.Calebiro@bham.ac.uk)).

**This file includes:**

Supplementary Figures 1-6

Source Data of Supplementary Figures 2 and 4


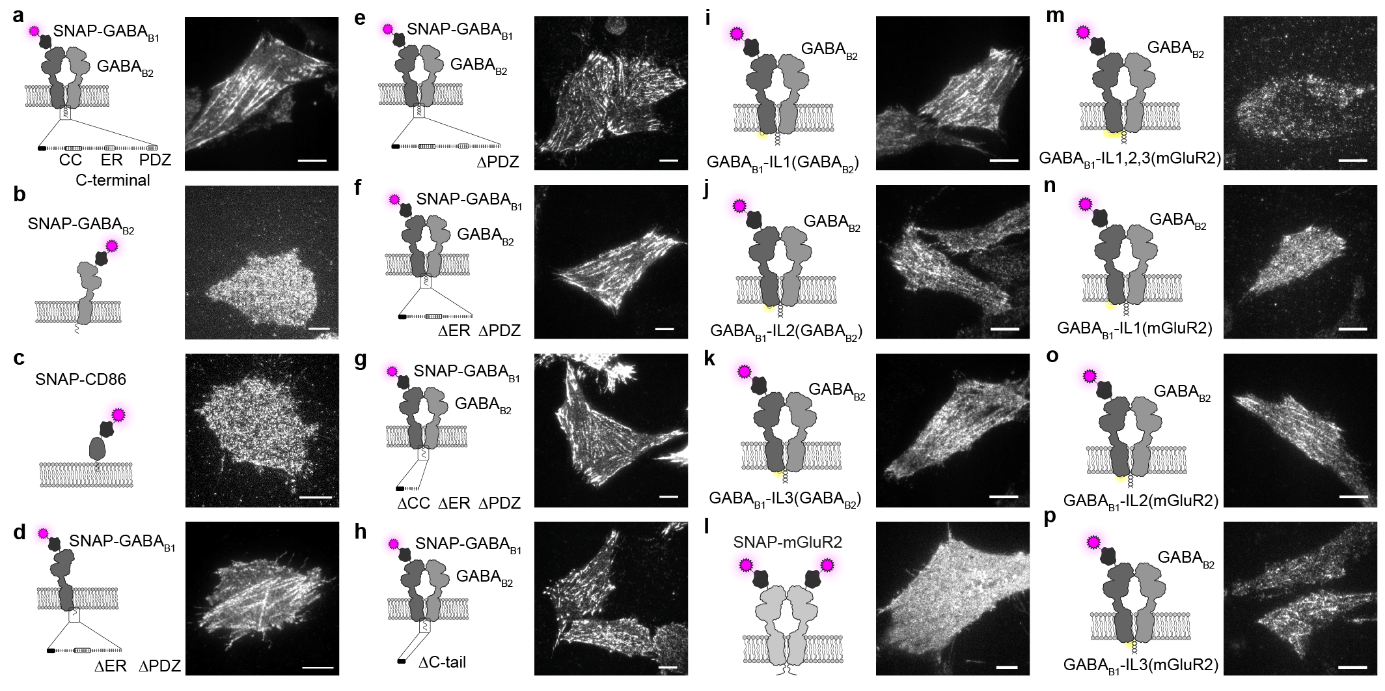


**Supplementary Figure 1.** **Cell surface organization of GABA_B_ mutants in CHO cells.** Unless otherwise stated, SNAP-tagged GABA_B1_ or GABA_B2_ subunits were co-transfected with the complementary wild-type GABAB_B1/B2_ subunit to allow the formation of GABA_B_ heterodimers and their correct cell-surface expression. Shown are representative TIRF images of SNAP-GABA_B1_ (**a**), SNAP-GABA_B2_ expressed alone (**b**), SNAP-CD86 (**c**), a SNAP-GABA_B1_ deletion mutant lacking the ER retention domain and PDZ motif (ΔER−ΔPDZ) expressed alone (**d**), a SNAP-GABA_B1_ deletion mutant lacking the C-terminal PDZ motif (ΔPDZ) co-expressed with GABA_B2_ (**e**), a SNAP-GABA_B1_ deletion mutant additionally lacking the ER retention motif (ΔER/ΔPDZ) (**f**), a SNAP-GABA_B1_ deletion mutant additionally lacking the coiled-coil domain (ΔCC/ΔER/ΔPDZ) (**g**), a SNAP-GABA_B1_ deletion mutant in which the whole C-tail has been removed (ΔC-tail) (**h**), a SNAP-GABA_B1_ mutant in which the first intracellular loop (IL1) has been replaced with the IL1 of the GABA_B2_ subunit (IL1 GABA_B2_) (**i**), a SNAP-GABA_B1_ mutant in which the IL2 has been replaced with the IL2 of GABA_B2_ (IL2 GABA_B2_) (**j**), a SNAP-GABA_B1_ mutant in which the IL3 has been replaced with the IL3 of the GABA_B2_ subunit (IL3 GABA_B2_) (**k**), SNAP-mGluR2, given as a reference (**l**), a SNAP-GABA_B1_ mutant in which all three intracellular loops have been replaced with the corresponding IL1, IL2 and IL3 of mGluR2 (IL1,2,3 mGluR2) (**m**), a SNAP-GABA_B1_ mutant in which the IL1 has been replaced with the IL1 of mGluR2 (IL1 mGluR2) (**n**), a SNAP-GABA_B1_ mutant in which the IL2 has been replaced with the IL2 of mGluR2 (IL1 mGluR2) (**o**), a SNAP-GABA_B1_ mutant in which the IL3 has been replaced with the IL3 of mGluR2 (IL3 mGluR2) (**p**). Scale bars, 10 µm. Images are representative of at least two independent experiments.


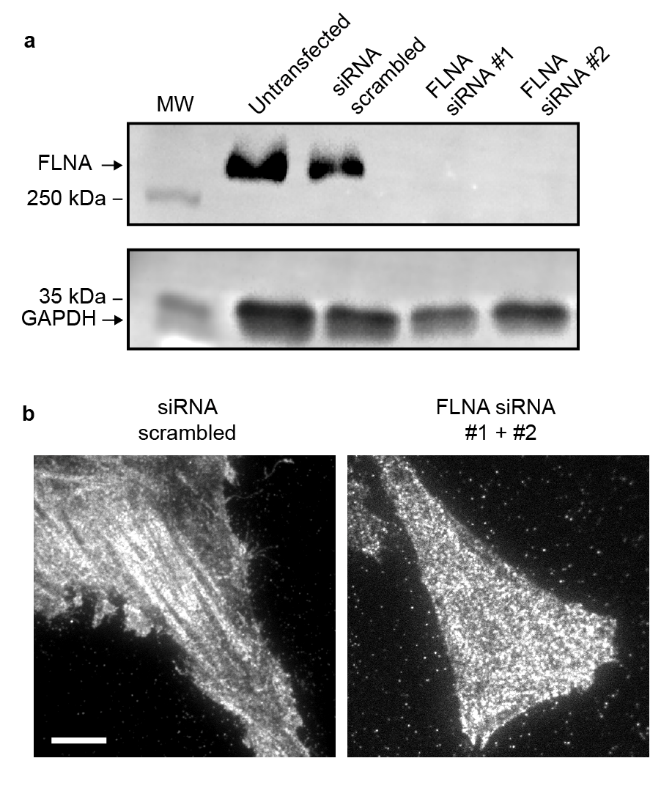


**Supplementary Figure 2.** **Effect of FLNA knockdown on GABA_B_ organization at the plasma membrane. a**, Western blot analysis showing the efficiency of FLNA knockdown in CHO cells. Lysates of untransfected cells or cells transfected with scrambled siRNA or two separate siRNAs (#1 and 2) against FLNA were probed with an anti-FLNA antibody. GAPDH was used as a loading control. MW, molecular weight. **b**, Representative TIRF images of CHO cells treated with either the scrambled siRNA or the two combined siRNAs against FLNA, followed by transfection with SNAP-GABA_B1_ and GABA_B2_ and labeled with SNAP-549. The images are representative of three independent experiments. Scale bar, 10 µm. Source data are provided as a Source Data file.


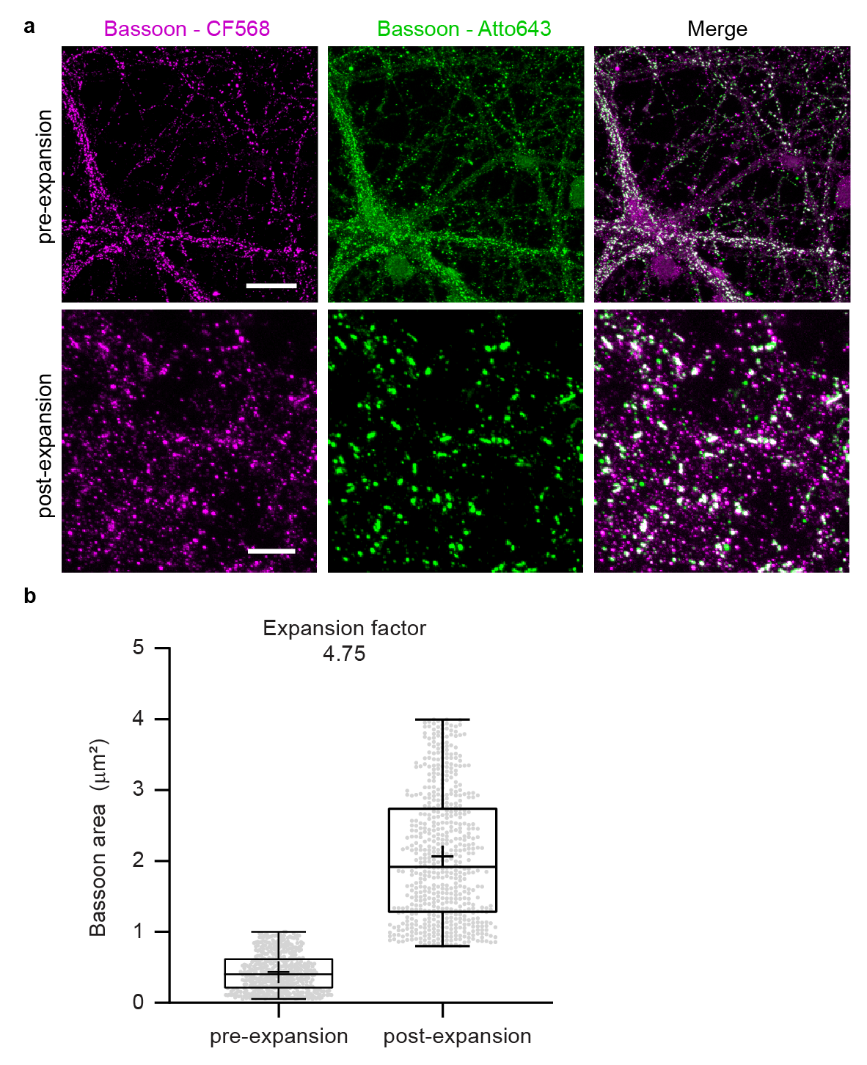


**Supplementary Figure 3**. **Expansion microscopy of Bassoon in hippocampal neurons. a,** Representative confocal microscopy images of fixed hippocampal neurons immunostained for Bassoon using two distinct antibodies (magenta and green) pre- and post-expansion. The corresponding MCC analyses are shown in Fig. 2k. **b,** Boxplot displaying bassoon area measured in images like in **a** before and after expansion microscopy allowing quantification of the expansion factor. Boxplot show lower to upper quartile and median values of the data with whiskers representing min to max values. Data are from three independent experiments. Mean values are represented by a cross on the plot. Scale bars, 20 μm (upper panel) and 10 μm (lower panel). Source data are provided as a Source Data file.


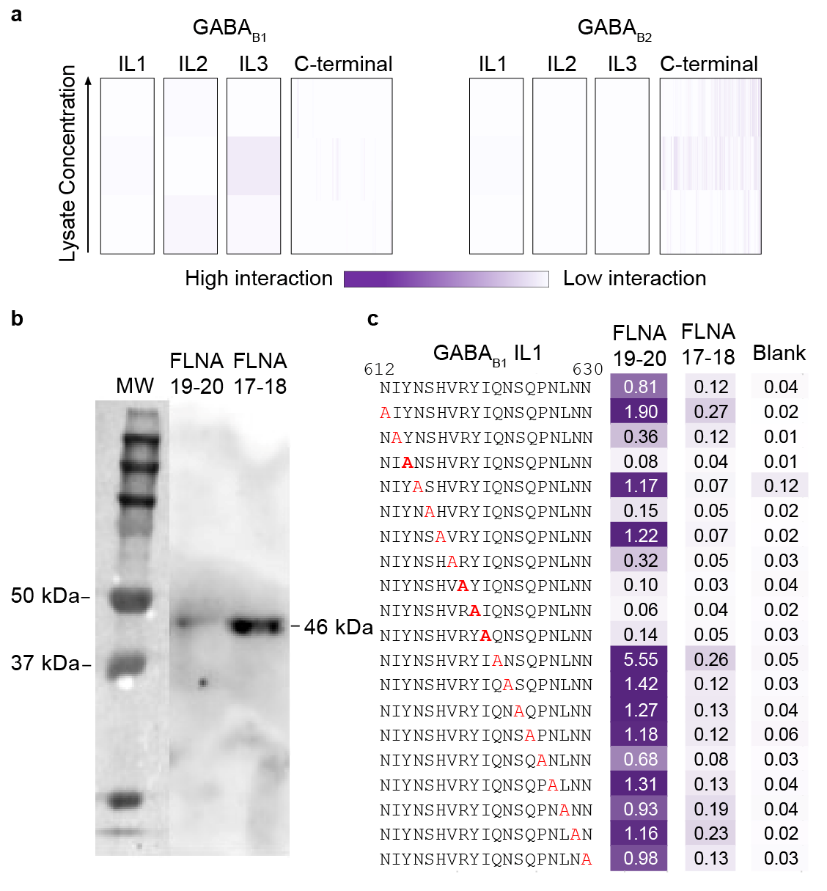


**Supplementary Figure 4**. **Specificity of peptide microarray binding assay.** **a**, Peptide microarrays corresponding to GABA_B1_ and GABA_B2_ intracellular domains were incubated with increasing concentrations of cell lysates containing the DsRed-FLNA17-18 fragment. Fluorescence readouts are plotted as a heatmap, normalized to the strongest signal detected with DsRed-FLNA19-20 (Fig. 3c).  **b,** Western blot analysis of cell lysates expressing DsRed-FLNA19-20 or DsRed-FLNA17-18 probed with an antibody against DsRed representative from two independent experiments. MW, molecular weight. **c,** Intensity values of the peptide microarray analysis shown in Fig. 3. Source data are provided as a Source Data file.


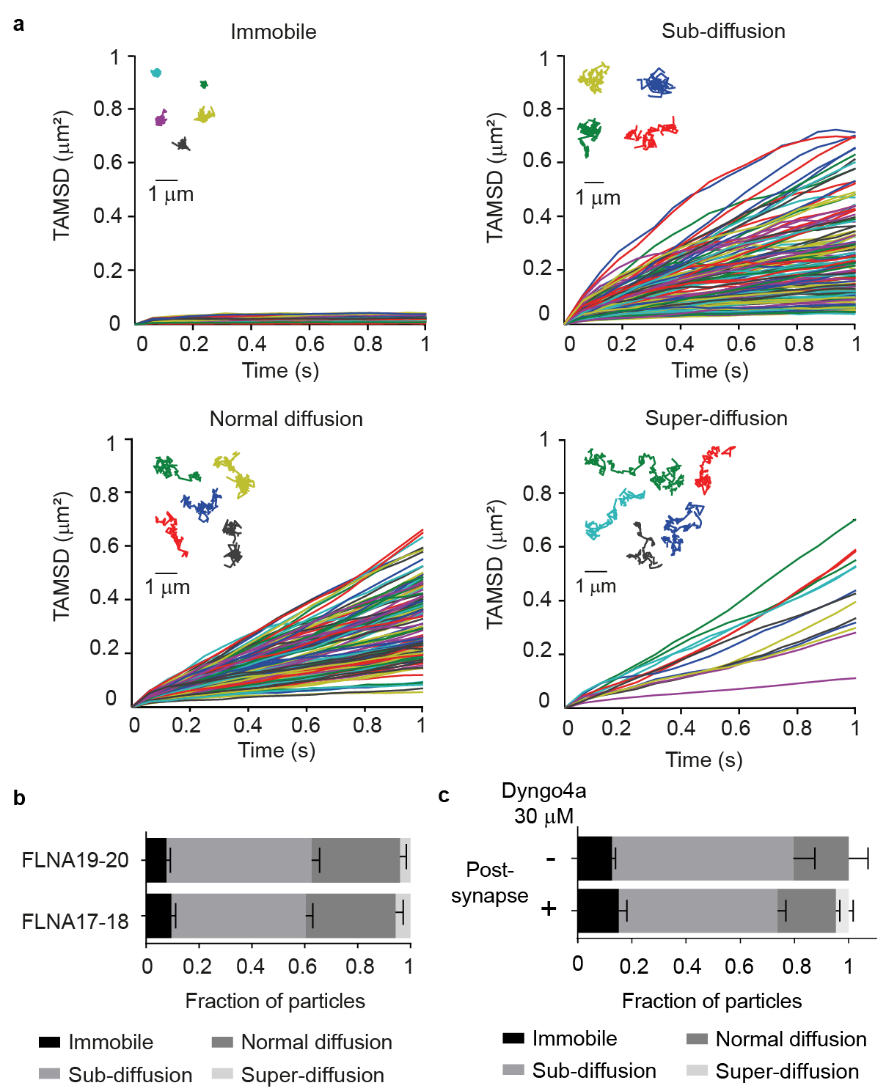


**Supplementary Figure 5. Time-averaged mean square displacement (TAMSD) analysis.** Individual GABA_B_ trajectories were separated into four categories based on their diffusion coefficient (*D*) and anomalous coefficient (*α*) (see Materials and methods for details). a, example of trajectories and corresponding TAMSD plots. **b**, Frequency distributions of GABA_B1_ trajectories in CHO cells classified in the four groups in the presence of either DsRed-FLNA17-18 or DsRed-FLNA19-20. Data are mean ± SEM. n=16 and 15 cells (2,809 and 2,670 trajectories) for FLNA17-18 and FLNA19-20, respectively, examined over three independent experiments. **c,** Frequency distributions of GABA_B1_ trajectories in hippocampal neurons pretreated with Dyngo4a (30µM) and stimulated for 5 min with GABA (100 µM), classified in the four diffusivity groups. Data are mean ± SEM of n=4 and 14 cells (236 and 255 trajectories) pretreated without or with Dyngo4a, respectively, examined over three independent experiments. Source data are provided as a Source Data file.


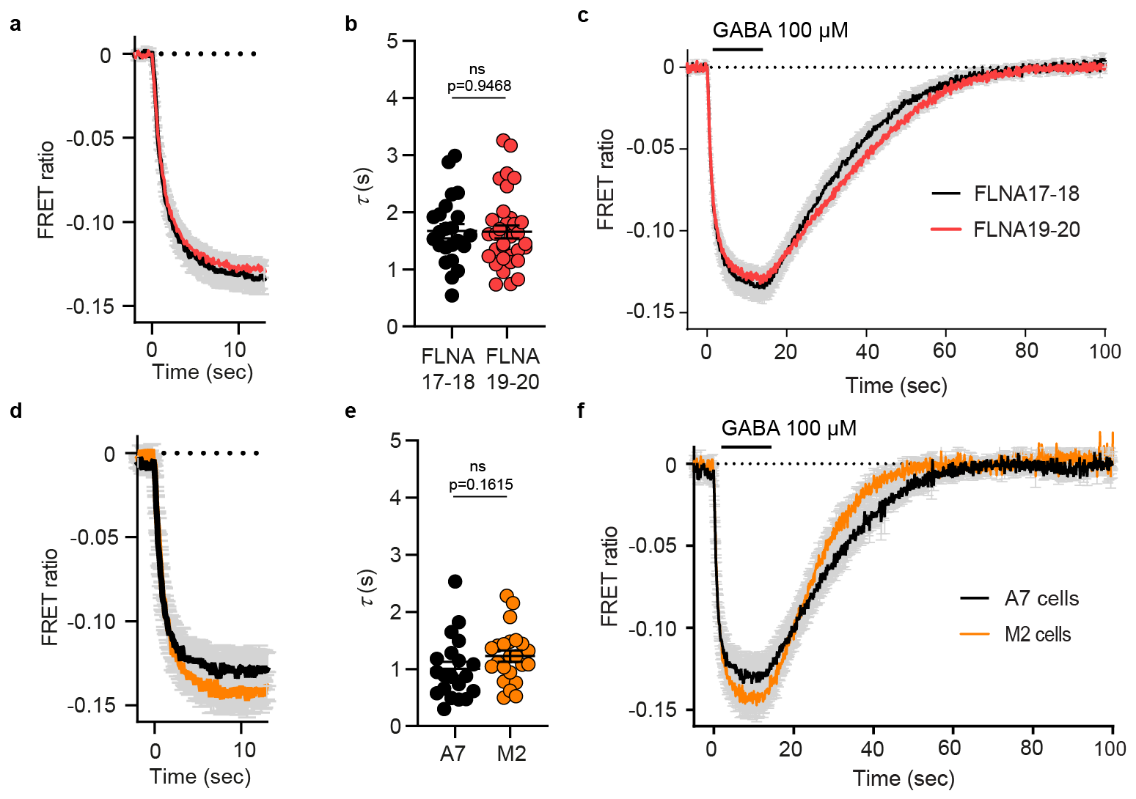


**Supplementary Figure 6.** **Kinetics of G_i_ protein activation and deactivation upon transient GABA_B_ stimulation. a,** Comparison of the kinetics of G_i_ protein activation during transient GABA_B_ receptor stimulation in HEK293A cells expressing either DsRed-FLNA17-18 or DsRed-FLNA19-20. **b,** Corresponding estimated time constants (*τ*) for G_i_ protein activation. ns, statistically not significant by two-tailed unpaired Welch’s t-test. **c,** Full time courses of G protein activation and deactivation in HEK293A cells expressing either DsRed-FLNA17-18 or DsRed-FLNA19-20 after transient stimulation with GABA. Data in **a**-**c** are mean ± SEM of n=22 and 30 cells for FLNA17-18 and FLNA19-20, respectively, examined over three independent experiments. **d,** Comparison of the kinetics of G_i_ protein activation in A7 and M2 cells after transient stimulation with GABA as in **a**. **e,** Corresponding estimated time constants (*τ*) for G_i_ protein activation. ns, statistically not significant by two-tailed unpaired Welch’s t-test. **f,** Full time courses of G protein activation and deactivation in HEK293A cells in M2 and A7 cells after transient stimulation with GABA. Data in **d**-**f** are mean ± SEM of n=20 and 23 cells for A7 and M2 cells, respectively, examined over three independent experiments. Source data are provided as a Source Data file.

**Source data of western blot gels**

**Source data of Supplementary Figure 2**


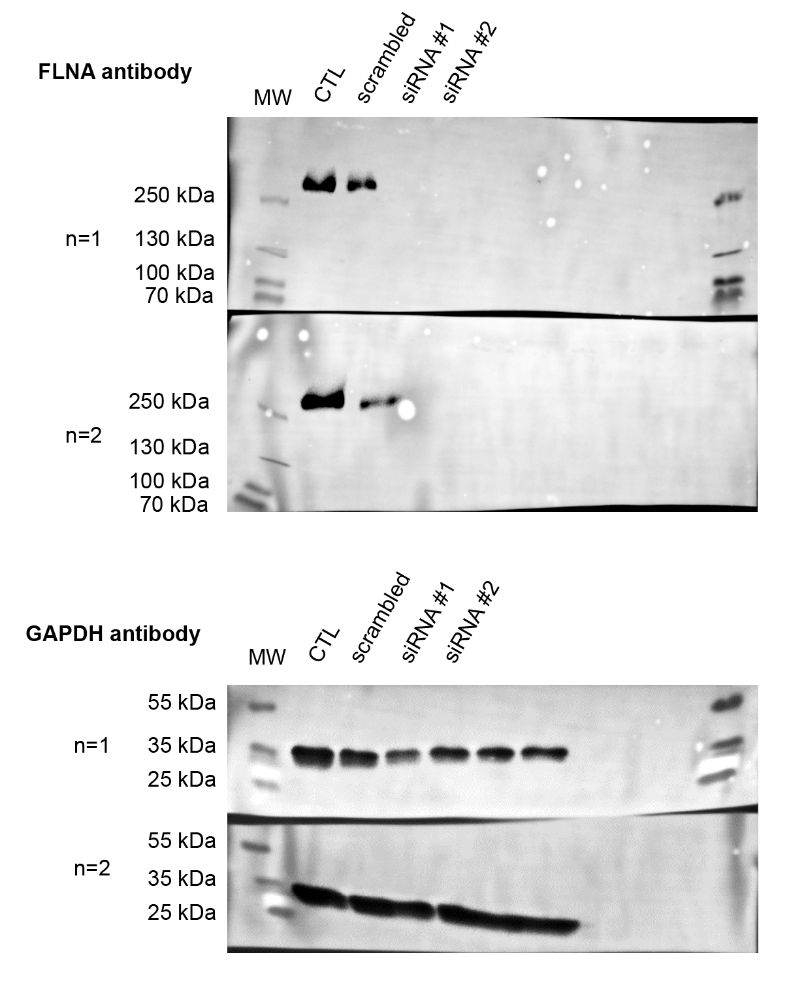


**Source data of Supplementary Figure 4**


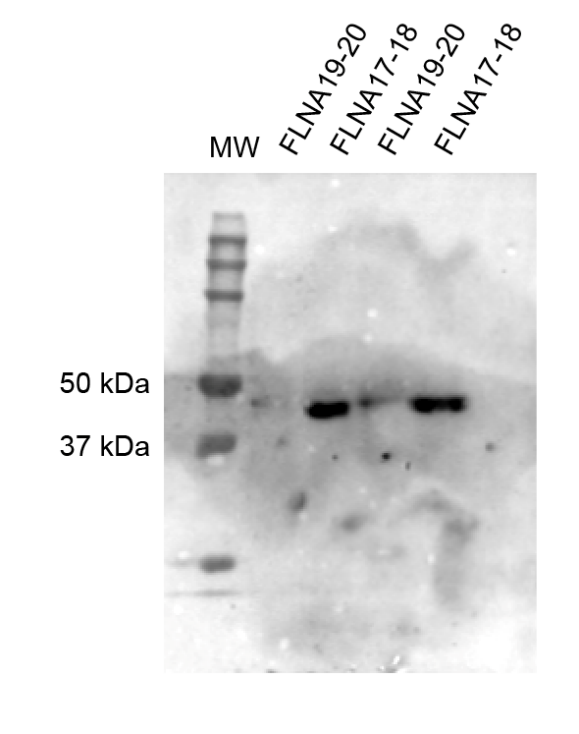

Supplement: Supplementary file 1 — Supplementary Information [file 41467_2022_35708_MOESM1_ESM.docx]
