# Supplementary material for: Filamin A organizes γ‑aminobutyric acid type B receptors at the plasma membrane
Source: Nat Commun. 2023 Jan 3;14:34. doi: 10.1038/s41467-022-35708-1 (PMC9810740; doi:10.1038/s41467-022-35708-1)
Supplement: Supplementary file 3 — Description of Additional Supplementary Files [file 41467_2022_35708_MOESM3_ESM.pdf]

Description of Additional Supplementary Files

File name: Supplementary Movie 1
Description: Single particle detection and tracking of $GABA_B$ receptors at the surface of CHO cells. Shown are the results of single particle tracking of $GABA_B$ receptors at the surface of CHO cells. Each particle detected is surrounded by a blue circle and trajectories are shown in magenta. Frames were acquired every 28 ms.

File name: Supplementary Movie 2
Description: Single-molecule imaging of $GABA_B$ receptors at the surface of CHO cells.
Shown are the results of single particle tracking of $GABA_B$ receptors (magenta) overlaid on a TIRF image of actin fibers (green). The tracked particles are surrounded by a blue circle and particle trajectories are shown in magenta. Frames were acquired every 28 ms.

File name: Supplementary Movie 3
Description: $GABA_B$ receptors transiently stopping along actin fibers.
Shown is a single molecule TIRF movie of an individual $GABA_B$ receptor (magenta) overlaid on actin fibers (green). Particle trajectories are shown in magenta. Frames were acquired every 28 ms.

File name: Supplementary Movie 4
Description: Two-color imaging of $GABA_B$ and FLNA at the surface of CHO cells.
Shown are individual trajectories of $GABA_B$ receptors (magenta) and FLNA (green) at the surface of CHO cells, imaged by fast two-color TIRF microscopy. Frames were acquired every 28 ms.

File name: Supplementary Movie 5
Description: Single-molecule imaging of $GABA_B$ receptors at the surface of hippocampal neurons.
Shown are the results of single particle tracking of $GABA_B$ receptors at the surface of hippocampal neurons. Each detected particle is surrounded by a blue circle and trajectories are shown in magenta. Frames were acquired every 28 ms.
