# Supplementary material for: Filamin A organizes γ‑aminobutyric acid type B receptors at the plasma membrane
Source: Nat Commun. 2023 Jan 3;14:34. doi: 10.1038/s41467-022-35708-1 (PMC9810740; doi:10.1038/s41467-022-35708-1)
Supplement: Supplementary file 9 — Reporting Summary [file 41467_2022_35708_MOESM9_ESM.pdf]

nature portfolio

Corresponding author(s): Davide Calebiro, Marie-Lise Jobin

Last updated by author(s): Nov 14, 2022

Reporting Summary

Nature Portfolio wishes to improve the reproducibility of the work that we publish. This form provides structure for consistency and transparency in reporting. For further information on Nature Portfolio policies, see our Editorial Policies and the Editorial Policy Checklist.

Statistics

For all statistical analyses, confirm that the following items are present in the figure legend, table legend, main text, or Methods section.

n/a	Confirmed	
☐	☒	The exact sample size (*n*) for each experimental group/condition, given as a discrete number and unit of measurement
☐	☒	A statement on whether measurements were taken from distinct samples or whether the same sample was measured repeatedly
☐	☒	The statistical test(s) used AND whether they are one- or two-sided<br>*Only common tests should be described solely by name; describe more complex techniques in the Methods section.*
☒	☐	A description of all covariates tested
☐	☒	A description of any assumptions or corrections, such as tests of normality and adjustment for multiple comparisons
☐	☒	A full description of the statistical parameters including central tendency (e.g. means) or other basic estimates (e.g. regression coefficient) AND variation (e.g. standard deviation) or associated estimates of uncertainty (e.g. confidence intervals)
☐	☒	For null hypothesis testing, the test statistic (e.g. *F*, *t*, *r*) with confidence intervals, effect sizes, degrees of freedom and *P* value noted<br>*Give P values as exact values whenever suitable.*
☒	☐	For Bayesian analysis, information on the choice of priors and Markov chain Monte Carlo settings
☒	☐	For hierarchical and complex designs, identification of the appropriate level for tests and full reporting of outcomes
☒	☐	Estimates of effect sizes (e.g. Cohen's *d*, Pearson's *r*), indicating how they were calculated

Our web collection on statistics for biologists contains articles on many of the points above.

Software and code

Policy information about availability of computer code

Data collection	TIRF images were acquired with NIS-Elements (Nikon). Confocal images were acquired with ZEN 12.0.1.362 (Zeiss). FRET data were acquired with Clampex 10.3 (Axon Instruments).
Data analysis	Statistical analyses were performed with GraphPad Prism 6.0. Microscopy images were analyzed using Fiji version 1.51p. Single-particle tracking was performed in MATLAB R2012a using u-track (Jaqaman K. et al., Nat. Methods 5, 695–702).<br>Key custom scripts used in the manuscript are available on the GitHub repository : https://github.com/CalebiroLab/GABAB_FLNA

For manuscripts utilizing custom algorithms or software that are central to the research but not yet described in published literature, software must be made available to editors and reviewers. We strongly encourage code deposition in a community repository (e.g. GitHub). See the Nature Portfolio guidelines for submitting code & software for further information.

Data

Policy information about availability of data

All manuscripts must include a data availability statement. This statement should provide the following information, where applicable:

- Accession codes, unique identifiers, or web links for publicly available datasets
- A description of any restrictions on data availability
- For clinical datasets or third party data, please ensure that the statement adheres to our policy

Source data are provided with this paper. Because of their large size (more than 1 Tb), the raw microscopic images underlying the results of our manuscript are available upon request to the corresponding authors.

March 2021

Field-specific reporting

Please select the one below that is the best fit for your research. If you are not sure, read the appropriate sections before making your selection.

☒ Life sciences ☐ Behavioural & social sciences ☐ Ecological, evolutionary & environmental sciences

For a reference copy of the document with all sections, see nature.com/documents/nr-reporting-summary-flat.pdf

Life sciences study design

All studies must disclose on these points even when the disclosure is negative.

Sample size	No statistical methods were used to predetermine sample size. Sample size was chosen based on previously published research (Sungkaworn et al., 2017, Treppiedi et al, 2018; Gentsch et al., 2020).
Data exclusions	No data were excluded.
Replication	All findings were replicated as specified in the figure legends. When representative data are shown, the number of times that the experiment was repeated with similar results is stated in the figure legend.
Randomization	The experiments were not randomized. Single-molecule data were analyzed by fully automated scripts with no user intervention during the analysis.
Blinding	TIRF images in figure 2d were acquired blindly. For single-molecule analyses, blinding was not needed as data were analyzed by fully automated scripts with no user intervention during the analysis.

Reporting for specific materials, systems and methods

We require information from authors about some types of materials, experimental systems and methods used in many studies. Here, indicate whether each material, system or method listed is relevant to your study. If you are not sure if a list item applies to your research, read the appropriate section before selecting a response.

Materials & experimental systems

n/a	Involved in the study
☐	☒ Antibodies
☐	☒ Eukaryotic cell lines
☒	☐ Palaeontology and archaeology
☐	☒ Animals and other organisms
☒	☐ Human research participants
☒	☐ Clinical data
☒	☐ Dual use research of concern

Methods

n/a	Involved in the study
☒	☐ ChIP-seq
☒	☐ Flow cytometry
☒	☐ MRI-based neuroimaging

Antibodies

Antibodies used	Rabbit anti-GABAB1a/b polyclonal antibody (B17) was provided by Ryuichi Shigemoto (Institute of Science and Technology, Austria).<br>Mouse Filamin A monoclonal antibody (M01) was purchased from Abnova (cat number: H00002316-M01), clone 4E10-1B2.<br>Mouse monoclonal anti-bassoon was from Enzo Life Sciences (cat number: ADI-VAM-PS003), clone SAP7F407.<br>Rabbit polyclonal anti-bassoon was from Synaptic Systems (cat number: 141 003).<br>Mouse GAPDH monoclonal antibody was from Invitrogen (cat number: AM4300), clone 6C5.<br>Mouse DsRed monoclonal antibody was from Santa Cruz (cat number: sc-390909), clone E-8.<br>Mouse IgG kappa binding protein coupled to HRP was from Santa Cruz (cat number: sc-516102).<br>Alexa Fluor-532 Phalloidin was from Thermo Fischer Scientific (cat number A-22282).<br>Goat Alexa Fluor-532 anti-mouse was from Thermo Fisher Scientific (cat number A-11002).<br>Goat Alexa Fluor-647 anti-rabbit was from Thermo Fisher Scientific (cat number A-21245).<br>Goat anti-rabbit IgG antibody was from Sigma (cat number: AP132).<br>Goat anti-rabbit CF568 was from Biotium (cat number: 20801).
Validation	Rabbit anti-GABAB1a/b polyclonal antibody (B17) was validated in Kulik Á. et al. Eur. J. Neurosci. 2002, 15, 291–307, doi:10.1046/j.0953-816x.2001.01855.x.<br>Mouse monoclonal anti-Filamin A antibody (M01) is verified by Abnova using western blot against the immunogen in HL-60 cells.<br>Mouse monoclonal anti-bassoon is verified by Enzo life Sciences using immunohistochemistry in the rat brain and western blot detection.<br>Mouse GAPDH monoclonal antibody was verified by western blot by ThermoFischer in various heterologous cells (COS-7, MDCK, PC-12 cells).<br>Mouse DsRed monoclonal antibody was validated by Santa Cruz by western blot on purified recombinant DsRed.

Mouse IgG kappa binding protein coupled to HRP was verified by Santa Cruz on various cell lysates.
Rabbit polyclonal anti-bassoon is validated by Synaptic Systems by immunocytochemistry in rat hippocampal neurons.
Secondary antibodies from Thermo Fischer Scientific were validated by immunocytochemistry on fixed cells.

Eukaryotic cell lines

Policy information about cell lines

Cell line source(s)

CHO-K1 cells were from Leibnitz-Institut DSMZ and HEK293A cells were from ATCC. The human M2 melanoma cell line and the stable subclone A7, were kindly gifted to Prof. Mantovani by Prof. Nakamura (Brigham and Women's Hospital, Boston, MA). These cells are commercially available from ATCC.

Authentication

Cells were cultured for a maximum of 20 passages. M2 and A2 cells were partially authenticated by morphology and immunostaining with a FLNA antibody. Commercial cell lines were not further authenticated.

Mycoplasma contamination

Cells were routinely tested for mycoplasma contamination by PCR using specific primers. All cell lines tested negative for mycoplasma contamination.

Commonly misidentified lines
(See ICLAC register)

None of the used cell lines is listed in the International Cell Line Authentication Committee registry (v.11).

Animals and other organisms

Policy information about studies involving animals; ARRIVE guidelines recommended for reporting animal research

Laboratory animals

Wild-type FVB and C57Bl/6J mice (male and female, 6-12 wks). Animals were housed under a 12 hour light/dark cycle at normal room temperature (22°C) and humidity 40-70% (typically 60%) with unrestricted access to food and water.

Wild animals

No wild animals were used in the present study.

Field-collected samples

No field-collected samples were used in the present study.

Ethics oversight

All animal work was done according to regulations of the relevant authority, the government of Lower Franconia, Bavaria and in accordance with the European guidelines for the care and use of laboratory animals, and the guidelines issued by the University of Bordeaux animal experimental committee.

Note that full information on the approval of the study protocol must also be provided in the manuscript.
